# Reorganization of the Social Brain in Individuals with Only One Intact Cerebral Hemisphere

**DOI:** 10.3390/brainsci11080965

**Published:** 2021-07-22

**Authors:** Dorit Kliemann, Ralph Adolphs, Lynn K. Paul, J. Michael Tyszka, Daniel Tranel

**Affiliations:** 1 Department of Psychological and Brain Sciences, University of Iowa, Iowa City, IA 52242, USA; daniel-tranel@uiowa.edu; 2 Department of Psychiatry, University of Iowa, Iowa City, IA 52242, USA; 3 Iowa Neuroscience Institute, University of Iowa, Iowa City, IA 52242, USA; 4 Division of Humanities and Social Sciences, California Institute of Technology, Pasadena, CA 91125, USA; radolphs@hss.caltech.edu (R.A.); lkpaul@hss.caltech.edu (L.K.P.); jmt@caltech.edu (J.M.T.); 5 Division of Biology and Bioengineering, California Institute of Technology, Pasadena, CA 91125, USA; 6 Department of Neurology, University of Iowa, Iowa City, IA 52242, USA

**Keywords:** emotion, social cognition, hemispherectomy, theory of mind, fMRI, plasticity

## Abstract

Social cognition and emotion are ubiquitous human processes that recruit a reliable set of brain networks in healthy individuals. These brain networks typically comprise midline (e.g., medial prefrontal cortex) as well as lateral regions of the brain including homotopic regions in both hemispheres (e.g., left and right temporo-parietal junction). Yet the necessary roles of these networks, and the broader roles of the left and right cerebral hemispheres in socioemotional functioning, remains debated. Here, we investigated these questions in four rare adults whose right (three cases) or left (one case) cerebral hemisphere had been surgically removed (to a large extent) to treat epilepsy. We studied four closely matched healthy comparison participants, and also compared the patient findings to data from a previously published larger healthy comparison sample (n = 33). Participants completed standardized socioemotional and cognitive assessments to investigate social cognition. Functional magnetic resonance imaging (fMRI) data were obtained during passive viewing of a short, animated movie that distinctively recruits two social brain networks: one engaged when thinking about other agents’ internal mental states (e.g., beliefs, desires, emotions; so-called Theory of Mind or ToM network), and the second engaged when thinking about bodily states (e.g., pain, hunger; so-called PAIN network). Behavioral assessments demonstrated remarkably intact general cognitive functioning in all individuals with hemispherectomy. Social-emotional functioning was somewhat variable in the hemispherectomy participants, but strikingly, none of these individuals had consistently impaired social-emotional processing and none of the assessment scores were consistent with a psychiatric disorder. Using inter-region correlation analyses, we also found surprisingly typical ToM and PAIN networks, as well as typical differentiation of the two networks (in the intact hemisphere of patients with either right or left hemispherectomy), based on idiosyncratic reorganization of cortical activation. The findings argue that compensatory brain networks can process social and emotional information following hemispherectomy across different age levels (from 3 months to 20 years old), and suggest that social brain networks typically distributed across midline and lateral brain regions in this domain can be reorganized, to a substantial degree.

## 1. Introduction

Thinking about other people’s internal states, such as their beliefs or emotions, is necessary for successful social interaction. Recognizing whether an approaching stranger is in a friendly or aggressive state, whether your child is in pain or in fear, or whether someone’s intentions are good or not, are crucial skills that develop over the first years of life. Almost instantly after being born, infants react to emotional information in their environment, such as the soothing sound of a parent’s voice [1]. People’s expressions of emotions in faces and body postures guide early behavior in infants [2,3], long before language has developed to communicate internal states verbally. The ability to infer other people’s beliefs and intentions (Theory of Mind, ToM) from their observed behavior—going beyond more basic emotion recognition—develops in children around the age of five [4]. Impairments in processing socioemotional information are common in affective disorders, such as depression [5], and are at the core of many neurodevelopmental disorders, such as autism [6]. Recent non-invasive functional neuroimaging work in children suggests that by age three, two core brain networks are functionally distinct: one for representing information about other people’s bodies (such as whether they are in pain or not), and another for representing information about their minds (such as what they believe) [7]. The division of labor between these two brain systems can be detected using fMRI even before behavioral milestones, such as passing false belief tasks, are typically achieved. The social brain networks underlying these inferences include midline as well as more lateral regions of the brain across both hemispheres.

Much of the functional organization of human cognition follows a largely symmetrical homotopic distribution across both cerebral hemispheres. Large-scale functional networks have been studied in thousands of healthy individuals using externally driven task activations as well as spontaneous fluctuations of brain activity “at rest” (rsfMRI, [8]). The majority of the identified functional networks (including primary sensory (e.g., visual, auditory) as well as higher cognitive (e.g., attention, psychomotor)) are typically distributed symmetrically across the left and right hemispheres. Even in the complete absence of direct structural connection via the corpus callosum, as demonstrated strikingly in patients with agenesis of the corpus callosum, the distribution of resting-state functional networks still follows a robustly homotopic organization [9,10]. Given the absence of one cerebral hemisphere, this homotopic distribution of functional rsfMRI networks is surprisingly intact and resembles typical spatial organization [11]. These findings confirm the substantial potential for plasticity and reorganization in the human brain when structural lesions occur early in life [12].

Yet there are also well-known exceptions to the symmetrical functional architecture in the brain (e.g., left lateralization of language). Regarding the social brain, a broad hypothesis suggests that the right hemisphere is more critical for socioemotional functioning than the left hemisphere. Support for lateralized social information processing has come from task-based fMRI studies [13]. Sensitivity to social information such as faces (right fusiform face area (FFA [14,15]), in occipital face area (OBA [16]), bodies (extrastriate body area, EBA [14,17]), biological motion (posterior superior temporal sulcus (pSTS [18]), and mental states (temporo-parietal junction, TPJ [19]) seem to be more prominent in the right hemisphere of the brain. These activation-based findings in healthy individuals are sometimes corroborated by causal perturbations: lesions [20] or experimental electrical stimulation [21] in the FFA also affect face perception in a right lateralized manner. However, sometimes lesion and fMRI evidence diverges: whereas fMRI studies argue for theory of mind processing subserved by the right TPJ, some lesion studies instead point to the left TPJ [22]. Furthermore, while multiple brain regions may be activated in a given process in healthy individuals, not all of these regions may be associated with deficits when lesioned—for instance, while the amygdala is sometimes also activated during theory of mind processing [23], it is not essential [24].

These findings point to the view that brain regions subserve social and emotional processing not in isolation but as components of anatomically distributed networks. We apply this framework in the present study, capitalizing on the whole-brain field-of-view provided by fMRI and by extensive prior knowledge of networks from imaging studies in healthy individuals. We focus on two networks: the Theory of Mind (ToM) network, and the pain network. The ToM network, also sometimes referred to as the “mentalizing network” [25], comprises a set of brain regions involved when thinking about others’ mental states, such as beliefs, desires and emotions [26,27,28,29]: dorsal (d)/ventral (v)/middle (m) medial prefrontal cortex (MPFC), precuneus (PC), bilateral temporal-parietal junction (TPJ). The pain network, sometimes referred to as “pain matrix,” includes a set of brain regions recruited when thinking about others’ bodily states, such as hunger or pain [30,31,32,33]: anterior mid-cingulate cortex (AMCC), bilateral insula, middle frontal gyrus (MFG) and secondary somatosensory cortex (S2). These two networks represent functional divisions for processing mental versus physical internal states of other agents across classic controlled condition subtraction task designs [32], and more recently with passive viewing of animated movie stimuli [7,34]. When watching complex social interactions, participants spontaneously attend to agents’ internal socioemotional states. Using such approaches for studying relatively distinct cognitive processing has proven to be a powerful tool to study the neurobiology of cognitive processes in the human mind [35,36,37,38], especially for studying socially-related cognitive processes [38], and for comparing atypical samples [39,40]. Even in the absence of an explicit task instruction, watching naturalistic movie stimuli can highlight brain behavior relationships), with fewer than 5 min of data [41].

Although fMRI studies have revealed largely bilaterally-distributed functional networks that are involved in socioemotional processing, there is also strong evidence, across a large number of types of tasks and stimuli, that the right hemisphere is more critical in this regard than the left. Lesions to the right hemisphere, more so than lesions to their left homologues, can impair the recognition of emotion from faces [42], the emotional content of sentences [43], for emotional and prosodic expression [44], emotion recognition across a number of different types of stimuli [45], so much so that it has been hypothesized that the right hemisphere is necessary for nonverbal socioemotional communication [46] in a way that might be a counterpart to the left hemisphere’s better known role in verbal communication [47]. There is some evidence that the right hemisphere may be more critical than the left for socioemotional processing even in infants [3] and possibly even in other animals including birds [48].

**Figure 1:**
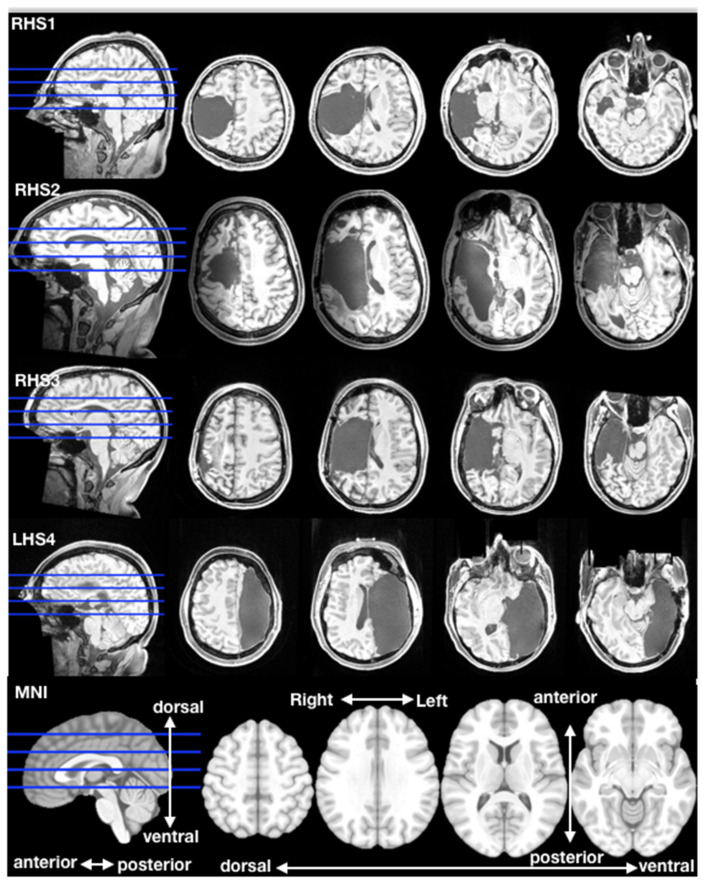
Axial sections of hemispherectomy brain anatomy. Three adult participants with right (RHS1, RHS2, RHS3) and one with left (LHS4) hemispherectomy. Four axial slices were taken in individual native space; axial inter-slice distance was 20 slices (1 mm slice thickness). Orientation legend is shown on the MNI template (bottom row).

The above brief survey suggests two key conclusions and an important open question. Socioemotional processing depends on distributed brain networks—such as the ToM and pain networks studied in the present report. While processing is normally distributed, lesion studies in neurological patients suggest a critical importance of the right hemisphere. What happens to the distributed networks following severe damage to the right hemisphere? The most dramatic case would be offered by patients with lesions of most or all of the right cerebral hemisphere. Might the absence of the right hemisphere prompt reorganization of cortical representation of social information to the left hemisphere? In the current study, we present data on rare individuals who underwent hemispherectomy surgery in childhood to treat intractable epilepsy [49]. Studying these patients offers a unique opportunity to combine lesions with functional neuroimaging to gain insight into reorganization. Hemispherectomy is a surgical procedure to isolate the affected hemisphere, either by removing it entirely (anatomical hemispherectomy, often also including all subcortical structures) or a partial anatomic resection and severing of all connections to the functional hemisphere (functional hemispherectomy [50]). Our multiple case study included three participants with right hemispherectomy and one participant with left hemispherectomy (Figure 1; Table 1). Although all of these individuals described some difficulties in everyday social interactions (e.g., few friends, difficulty interpreting non-verbal communication), they presented with strikingly normal social behavior during interactions with the investigators and during data acquisition within the context of this study (e.g., motivated, compliant, following directions accurately, engaging in small talk, laughing at jokes). To assess socioemotional processing in the brain, we acquired fMRI data while participants watched a short, animated movie with emotional content (“Partly Cloudy” [51]), which robustly distinguishes between the ToM and pain networks. We further included two comparison datasets: (1) Four healthy comparison participants scanned on the same scanner and with the same imaging sequence, closely matched in age, gender, handedness and basic levels of cognitive functioning (see Table 1). (2) Brain network data from a previously published independent sample of healthy participants who were imaged with the same movie stimulus (“Richardson et al.”, [7]). Building on our prior fMRI work in patients with hemispherectomy [11], we compared correlations of hemodynamic responses in a priori set brain regions when participants were passively watching the movie as an index of network connectivity (within network correlation) and distinctiveness (between network correlation) [35].

## 2. Materials and Methods

### 2.1. Participants

Three adults with right hemispherectomy (HS = hemispherectomy participants, 2 males; 3 right-handed; mean age = 22 ± 1 year), one adult with left hemispherectomy (female; left-handed; age 26) and four healthy adults (CNT = comparison participants, 2 males; 3 right-handed; mean age = 25.5 ± 1.91 years) participated in this study (Table 1). The right hemispherectomy and healthy comparison groups were similar with respect to overall intelligence (mean Full Scale Intelligence Quotient: right HS = 93.67 ± 12.66, CNT = 98.75 ± 3.20), as well as verbal and perceptual scores (mean Verbal Comprehension Index right HS = 100.33 ± 9.02, CNT = 103.5 ± 3.11; mean Perceptual Orientation Index right HS = 83.67 ± 10.69, CNT = 94.50 ± 1.91), and intelligence scores of the participant with left HS were within 1 standard deviation of the right HS group mean (see Table 1); HS tested with Wechsler Adult Intelligence Scale III [52], and comparison group with Wechsler Abbreviated Scales of Intelligence II [53]); all participants had completed high school. Right hemispherectomy resulted from a different etiology in each participant: Rasmussen’s encephalitis, cortical dysplasia, perinatal stroke. Left hemispherectomy resulted from perinatal stroke. Age at seizure onset ranged from birth to 11 years and age at surgery ranged from 3 months to 20 years. Three individuals underwent functional hemispherectomy, i.e., large sections of the affected hemisphere were resected and all connections of remaining tissue to the functional hemisphere were disconnected. One patient had a complete anatomical hemispherectomy (LHS4). Demographic information for all participants, as well as detailed neurological history related to hemispherectomy is provided in Table 1. Neurotypical comparison participants were recruited through the Caltech Conte Center, which applies the following exclusion criteria at enrollment: a first-degree relative with schizophrenia or autism spectrum disorder, currently taking psychotropic medication, uncorrected vision or hearing impairment, and moderate–severe depression or indication of current suicidality (Beck Depression Inventory–II total = 25+; score of 3 or 4 on item 9 [54]). Additional exclusionary criteria included history of any of the following: premature birth, epilepsy, major medical condition, metabolic disorder, chemotherapy or radiation, brain surgery, head injury, eating disorder, neurological condition, psychosis, bipolar disorder, autism, suicide attempt, substance dependence or abuse, alcoholism, color blindness or strabismus. With the exception of history of epilepsy and brain surgery, all of these exclusion criteria also applied to hemispherectomy participants. Participants were scanned at the Caltech Brain Imaging Center (CBIC, https://cbic.caltech.edu/, accessed on 15 July 2021) and signed written informed consent prior to participation in accordance with protocols approved by the Institutional Review Board of the California Institute of Technology.

### 2.2. Psychological and Behavioral Measures

Participants with hemispherectomy completed self-report questionnaires on the day of testing regarding the current presence of positive and negative emotions (Positive and Negative Affect Scales, PANAS [55]) and current anxiety (State-Trait Anxiety Inventory-State, STAI-S [56]). All participants completed self-report questionnaires regarding psychological traits including: trait anxiety (STAI-T), drive to empathize (empathizing quotient, EQ [57]), drive to analyze rule-based systems (systemizing quotient, SQ [58]), and deficits in social skills consistent with autism (Social Responsiveness Scale-2 Adult Self-Report, SRS-2 [59]). All participants with hemispherectomy and 2 comparison participants also completed a self-report and behavioral measure of ability to perceive and process emotions (Mayer-Salovey-Caruso Emotional Intelligence Test, MSCEIT [60]) that produces scores for experiencing and reasoning about emotions. For all measures, individual participant scores were converted to z-scores using published comparison group means (PANAS, [61], EQ and SQ, [62]), or standardized score conversion provided by the test publisher (STAI, SRS-2, MSCEIT). The scores reported below (see Results) thus indicate where an individual’s performance falls relative to published normative samples. These measures were used to screen for mood symptoms that might have interfered with attention during the MRI study (i.e., elevated state anxiety symptoms or highly polarized mood on the day of testing), evidence of ongoing elevations in anxiety (trait) or alterations in socioemotional functioning (e.g., symptoms consistent with an autism spectrum disorder; elevated SRS-2, or a pattern of elevated SQ and lowered EQ), or evidence of impairments in perception or reasoning about emotions (MSCEIT).

### 2.3. fMRI Stimulus

Participants passively watched a short, animated movie (“Partly Cloudy” [51], see https://www.pixar.com/partly-cloudy#partly-cloudy-1, accessed on 15 June 2021, for information on plot) during fMRI scanning and listened to the original movie audio over MR-compatible headphones (no speech, but prosodic vocalizations and background music). The movie has been used in previous studies with adults and children [7,31,32,63] and has been shown to evoke neural responses in brain regions involved when thinking about other peoples’ mental and physical internal states. Participants had no explicit task instructions other than to relax, watch the movie and to move as little as possible. All participants completed other tasks during the same scanning session that were not analyzed in this current study. Three of the four HS participants watched the movie twice, in different sessions across two days or on the same day. Attention to the stimuli was ensured by monitoring with an in-scanner camera directed at the participant’s eyes.

### 2.4. MRI Data Acquisition

Data from the hemispherectomy and matched comparison participants were acquired at the Caltech Brain Imaging Center (CBIC) using a 3 Tesla Magnetom Prisma MRI scanner (Siemens Medical Solutions, Erlangen, Germany). For each participant, we analyzed T1w structural data (MP-RAGE, TR/TE/TI = 1590 ms/2.7 ms/800 ms, 0.9 mm isotropic voxel size, flip angle = 10°) and T2*-weighted EPI functional data (TR = 700 ms, TE = 30 ms, flip angle = 53°, 2D multiband acquisition with 2.5 mm isotropic voxels). Geometric distortion corrections for the T2*-weighted EPI data were estimated from phase-encoding polarity reversed pairs of SE-EPI images (TR/TE = 5005/48 ms, flip angle = 90°) with identical geometry to the T2*-weighted EPI series.

### 2.5. MRI Preprocessing

Raw DICOM images were converted to Nifti-1 format files and organized according to the BIDS specification (http://bids.neuroimaging.io/, accessed on 15 July 2021) with the Docker container version of BIDSKIT (https://github.com/jmtyszka/bidskit), accessed on 15 July 2021. After conversion, minimal preprocessing was performed using FMRIPREP version 20.2.1, a Nipype-based tool [64].

### 2.6. Anatomical Data Preprocessing

The T1-weighted (T1w) image was corrected for intensity non-uniformity (INU) with *N4BiasFieldCorrection* [65], distributed with ANTs 2.3.3 [66], and used as T1w-reference throughout the workflow. The T1w-reference was then skull-stripped with a *Nipype* implementation of the *antsBrainExtraction.sh* workflow (from ANTs), using OASIS30ANTs as target template. Brain tissue segmentation of cerebrospinal fluid (CSF), white-matter (WM) and gray-matter (GM) was performed on the brain-extracted T1w using *fast* (FSL 5.0.9) [67]. Volume-based spatial normalization to the ICBM/MNI 152 nonlinear asymmetrical standard space (MNI152NLin2009cAsym [68]) was performed through nonlinear registration with *antsRegistration* (ANTs 2.3.3), using brain-extracted versions of both T1w reference and the T1w standard space template.

### 2.7. fmri Preprocessing

For each of the BOLD-fMRI runs obtained per subject (across all tasks and sessions), the following preprocessing was performed. First, a reference volume and its skull-stripped version were generated using a customized methodology of *fMRIPrep*. A B_0_ field map was estimated based on two echo-planar imaging (EPI) references with opposing phase-encoding directions, with *3dQwarp* (AFNI version 20160207) [69]. Based on the estimated susceptibility distortion, a corrected echo-planar imaging (EPI) reference was calculated for a more accurate co-registration with the anatomical reference. The BOLD reference was then co-registered to the T1w reference using *flirt* (FSL 5.0.9) [70,71,72] with the boundary-based registration cost-function [73].

Co-registration was configured with nine degrees of freedom to account for distortions remaining in the BOLD reference. Head motion parameters with respect to the BOLD reference (transformation matrices, and six corresponding rotation and translation parameters) were estimated before any spatiotemporal filtering using *mcflirt* (FSL 5.0.9) [70]. The BOLD time series were resampled onto their original, native space by applying a single, composite transform to correct for head motion and susceptibility distortions. These resampled BOLD time series will be referred to as preprocessed BOLD in original space. For the comparison subjects’ data only, the BOLD time series were resampled into standard space, generating a preprocessed BOLD series in MNI152NLin2009cAsym space. Several confounding time series were calculated based on the preprocessed BOLD series: framewise displacement (FD), DVARS and three region-wise global signals (WM, GM CSF). FD was computed using two formulations following Power (absolute sum of relative motions) [74] and Jenkinson (relative root mean square displacement between affines) [71]. FD and DVARS were calculated for each functional run, both using their implementations in *Nipype* following the definitions [74]. The head motion estimates calculated in the correction step were also placed within the corresponding confounds file. The confound time series derived from head motion estimates and global signals were expanded with the inclusion of temporal derivatives and quadratic terms for each [75]. All resampling was performed with a single interpolation step by composing all the pertinent transformations (i.e., head motion transform matrices, susceptibility distortion correction when available, and co-registrations to anatomical and output spaces). Gridded (volumetric) resampling was performed using *antsApplyTransforms* (ANTs), configured with Lanczos interpolation to minimize the smoothing effects of other kernels [76].

No slice timing correction was applied given the sub-second TR. Functional data were projected only onto each subject’s native anatomical space and not to template spaces to reduce distortion of atypical hemispherectomy anatomy.

### 2.8. fMRI Denoising

Non-neural signal components arising from breathing, heartbeat, head motion, and imaging artifacts were identified and removed using spatial independent component analysis (ICA) implemented by the MELODIC tool in the FSL software suite (FSL 5.10.0) [72,77,78]. ICA was performed in individual space following motion correction to minimize the impact of resampling errors following transformation to the standard space. Resulting independent components were inspected visually following standard guidelines [79]. Identified non-neural noise components were regressed from the data (*fsl_regfilt* in FSL), and a linear highpass filter (*fslmaths* in FSL, σ = 143 volumes) was applied to all denoised BOLD data prior to subsequent analysis.

### 2.9. Head Motion

Levels of head motion in functional runs were relatively low across HS participants given that clinical populations often exhibit greater levels of motion. While both groups showed comparable levels of head motion as measured with framewise displacement (FD) the highest level of motion was found in CNT1 with temporal mean FD = 0.3 mm, as compared to the highest value for a HS participant with temporal mean FD = 0.23 mm (see Table 2 for individual FD results). All other temporal mean FD values were well matched between cases.

### 2.10. Region of Interest (ROI) Definition

**Figure 2:**
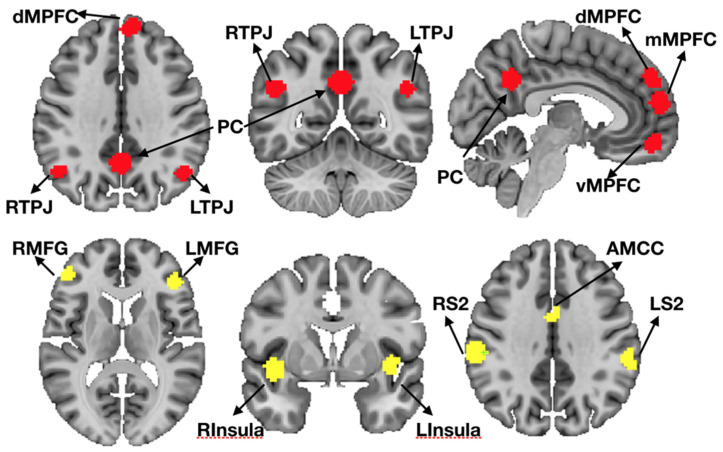
Regions of interest displayed on the MNI template (displayed in 2.5 mm space). Regions comprising the ToM network (upper row, in red): (dorsal (d)/ventral (v)/middle (m) medial prefrontal cortex (MPFC), precuneus (PC), right temporal-parietal junction (RTPJ), left temporal-parietal junction (LTPJ); and the “Pain Matrix” network (lower row, in yellow): anterior mid-cingulate cortex (AMCC), right insula (RInsula), left insula (LInsula), right middle frontal gyrus (RMFG), left middle frontal gyrus (LMFG), right secondary somatosensory cortex (RS2), left secondary somatosensory cortex (LS2)).

We used a set of a priori independently defined group ROIs (“Tom/Pain” ROIs) based on responses to the “Partly cloudy” movie in an independent sample, described in detail elsewhere (see [7]; available for download (https://www.openfmri.org/dataset/ds000228/, accessed on 15 July 2021)). In short, ROIs were defined on independent sample of participants defining 9mm spheres surrounding group peak activations to mental and pain events. These ROIs included six regions of the Theory of Mind network (dorsal (d)/ventral (v)/middle (m) medial prefrontal cortex (MPFC), precuneus (PC), bilateral temporal-parietal junction (TPJ)) and the “Pain Matrix” (anterior mid-cingulate cortex (AMCC), bilateral insula, middle frontal gyrus (MFG) and secondary somatosensory cortex (S2)) (Figure 2).

To compare social brain networks in the left and right hemispheres we created two lateralized sets of ROIs: For the left hemisphere, we masked all midline ROIs with a mask of the left hemisphere, effectively deleting any voxels originally in the right hemisphere. For the right hemisphere ROIs we adopted a different strategy because original prefrontal medial ROIs (d/m/vMPFC) were lateralized with an unequal distribution across the midline, resulting in a greater number of voxels in the left hemisphere for these ROIs. To avoid unbalanced numbers of voxels when comparing networks in only one hemisphere, midline ROIs for the right hemisphere were created by mirroring voxels from the left hemisphere into the right hemisphere. This procedure resulted in three sets of ROIs per network: (1) original midline and bilaterally symmetric ROIs, (2) lateralized ROIs in the left hemisphere (left TPJ, left insula, left S2, left MFG and voxels of the midline ROIs within the left hemisphere and (3) lateralized ROIs in the right hemisphere (right TPJ, right insula, right S2, right MFG and voxels of the midline ROIs within the right hemisphere plus voxels mirrored from the left hemisphere. For the comparison control participants (n = 4) with two intact hemispheres, we presented three sets of ROIs: (a) bilateral including both complete midline and lateral ROIs as well as (b) ROIs restricted to only the (b) left and (c) right cortical regions of ROIs.

### 2.11. ROI Registration Procedure

Registration of ROIs in the MNI152 2009c nonlinear symmetric space were mapped to the intact hemisphere of each hemispherectomy subject using the following approach. A mask was created manually for the intact hemisphere and the ipsilateral midbrain, brainstem and cerebellum at midline using ITK-SNAP [80]. The original T1w structural image was convolved with a smoothed version of the mask (Gaussian filter, σ = 0.5 mm) to remove all tissue outside the intact hemisphere but leave a smooth transition between non-zero and zero signal at the pial surface. Left and right single-hemisphere versions of the T1w MNI template were created by simple masking at midline of the brain-only template. The appropriate template (left or right) was registered to the masked intact hemisphere of each patient using successive multiscale, rigid body, affine and symmetric normalization diffeomorphic transforms implemented by the *antsRegistrationSyNQuick.sh* script in ANTS 2.2 [66]. The resulting template-to-individual transform was then used to map ROIs defined in the MNI standard space to the individual intact hemisphere of each patient. For comparison subjects, ROIs in MNI space were simply transformed into functional space (2.5 isotropic. ROIs were thresholded (0.5) and binarized post transform for both comparison and hemispherectomy subjects.

### 2.12. FMRI Inter-Region Correlation of Time Series Analysis

To assess to what extent regions of the ToM and Pain networks show synchronized responses, we extracted time series from ROIs and averaged across all voxels within an ROI (with *fslmeants* in FSL) per run for each subject. Averaged time series were correlated across ROIs and networks with Pearson’s correlation. Within (e.g., all ROIs in the Tom network) and between network (e.g., all ROIs in the Tom network and all ROIs from the Pain network) correlations were calculated within subjects and runs per ROI. Correlations were Fisher z transformed before any further calculation. If subjects had data for two runs, correlation matrices were averaged across runs. Code for this analysis was custom written in MATLAB (version 2019a, The MathWorks, Natick, MA, USA) and is available upon request.

### 2.13. Comparison Data Previously Published in Richardson et al., 2018

For a reference comparison of the presented individual HS patient and closely matched healthy control data, we included previously published within and between network correlation data for the network ROIs from 33 healthy participants (mean age 25 years, 32 right-handed, 20 females) from Richardson et al., 2018 [7]. Data preprocessing and sample characteristics are described in detail in the original publication. The main difference in preprocessing in this study is the inclusion of physiological denoising with CompCor, motion outlier time point exclusion (“scrubbing”), and spatial smoothing. Spatial smoothing in particular improves signal-to-noise at the expense of spatial resolution and may result in larger within- and between-network correlation differences. The sample from Richardson et al. helps establish that our findings in comparison participants are indeed comparable to previous reports using a conceptually similar analysis. Given the differences in preprocessing this study can be considered a “far replicability” study of Richardson et al. (see, e.g., [81]) showing relative independence from preprocessing differences. Data from the distant reference group is based on bilateral ROIs only.

### 2.14. Quantification of Results

This study was designed as a multiple case study, and we consequently do not present any group-level statistical analyses or multiple comparison corrections. Instead, we show effect sizes (strength of correlations), with attention to presenting all the individual data. 

## 3. Results

### 3.1. Behavioral Data

All participants scored within the average range (i.e., within 1.5 standard deviations from the normative sample mean) on both ability to experience and reason about emotions (MSCEIT). On self-report measures of current mood, anxiety, and social skills, the participant with left HS and all comparison participants scored within the average range (i.e., within 1.5 standard deviations from the normative sample mean), with the exception of a clinically significant elevation on trait anxiety in one comparison participant (CNT4). Self-report scores from the three participants with right HS did not reveal clinically significant symptoms of anxiety or social impairments, although there was some variability in symptoms across the three participants. Two participants with right HS (RHS1 & RHS2) reported elevated levels of empathy (>1.5 standard deviation from normative sample mean), which suggests high levels of empathy relative to the normative sample (and would not be consistent with restriction in measured empathy typically found in autism spectrum disorders). Ratings of positive mood were elevated for RHS1 and lowered for RHS3 by over 1.5 standard deviations from the normative sample mean. Both of these participants scored in average range for negative mood, suggesting that the atypical positive mood scores did not reflect a clearly polarized mood state that might interfere with the MRI task (see Table 3).

During data acquisition, the individuals with hemispherectomy behaved normally in virtually all respects: alert, attentive, highly motivated, and compliant with instructions for behavioral tasks and fMRI data acquisition. They communicated well, using age-appropriate expressive language with good articulation and exhibited no difficulty with language comprehension. With respect to social cognition, they were indistinguishable from individuals with two complete hemispheres: engaging in small talk, following common social rules and etiquette (e.g., laughing at experimenters’ jokes). Apart from obvious motor impairments in the arm and leg contralateral to the resection site, the behavioral impact of their hemispherectomy could go unnoticed in short, nonspecific interactions.

### 3.2. Functional MRI Data

#### Inter-Region Correlation Analysis

**Figure 3:**
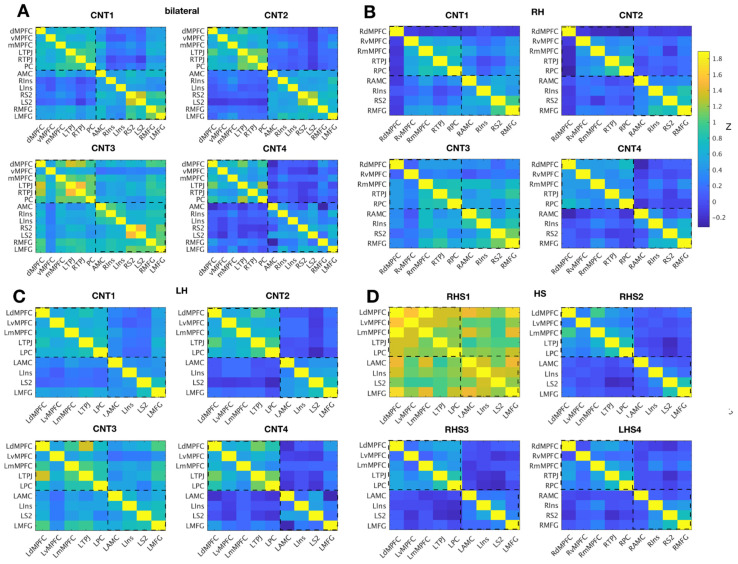
Correlation matrices indicating strength of correlations (Fisher’s z) per sample across regions of the ToM and PAIN network for the original (**A**), right lateralized (**B**) and left lateralized (**C**) sets of ROIs in the comparison participants (CNT), as well as HS participants (**D**). ToM network ROIs are listed at the top of the y axis and left on the x-axis: dMPFC = dorsal medial prefrontal cortex, vMPFC = ventral medial prefrontal cortex, mMPFC = middle medial prefrontal cortex, PC = precuneus PC, TPJ = bilateral temporal-parietal junction. PAIN network ROIs include: AMCC = anterior mid-cingulate cortex, ins = insula, MFG = middle frontal gyrus and S2 = secondary somatosensory cortex. Hemispheric lateralization of ROIs is indicated by R or L prior to the ROI abbreviation. Abbreviations: LH, left hemisphere; RH right hemisphere; CNT1-4, comparison individuals; RSH1-3 right hemispherectomy individuals; LHS4, left hemispherectomy individual.

**Figure 4:**
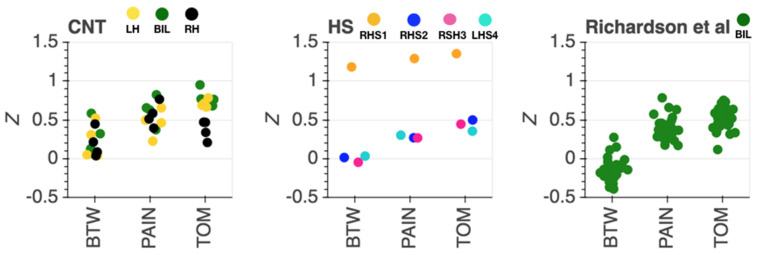
Strength of network connectivity (Fisher’s z) within (ToM, PAIN) and between (BTW) social brain networks for the hemispherectomy (HS, **middle**), comparison s (CNT, **left**) and a previously published independent dataset of comparison participants (Richardson et al., [7] **right**). Each datapoint corresponds to one subject. For CNT, type of ROI is color coded (bilateral: green; LH: yellow; RH: black), for HS, subjects are color coded (RHS1 orange, RHS2: blue, RHS3: pink, LHS4 turquoise), for Richardson et al. [7] comparison data, all data are from bilateral (green) ROIs per subject.

We conducted inter-region correlation analyses to investigate levels of synchronization within and between two social brain networks in hemispherectomy and comparison participants while participants watched an animated movie in the scanner: the Theory of Mind network (ToM), recruited when thinking about other people’s internal mental states, and the pain matrix (PAIN), recruited when thinking about other people’s physical states. We conducted two sets of comparisons:

First, as a control analysis, we tested whether we could replicate previously reported functional separation of the ToM and PAIN networks in the four healthy comparison participants. For this initial test, we used the original sets of ROIs including midline as well as bilateral ROIs (Figure 3a). On the single subject level, the four comparison participants all showed correlation of changes in hemodynamic responses to the movie *within* the two social brain networks (see Figure 3a). Correlations of brain regions *between* regions of the two networks were reduced for all four subjects as compared to regions within networks, yet to varying degrees (see Figure 4, middle). Interestingly, the only comparison subject who was left-handed (CNT3) showed overall less network dissociation (i.e., greater synchronization between regions that were from different networks). This effect seemed unlikely to be related to head motion, as CNT3 showed the least overall amount of head motion among the comparison subjects.

**Figure 5:**
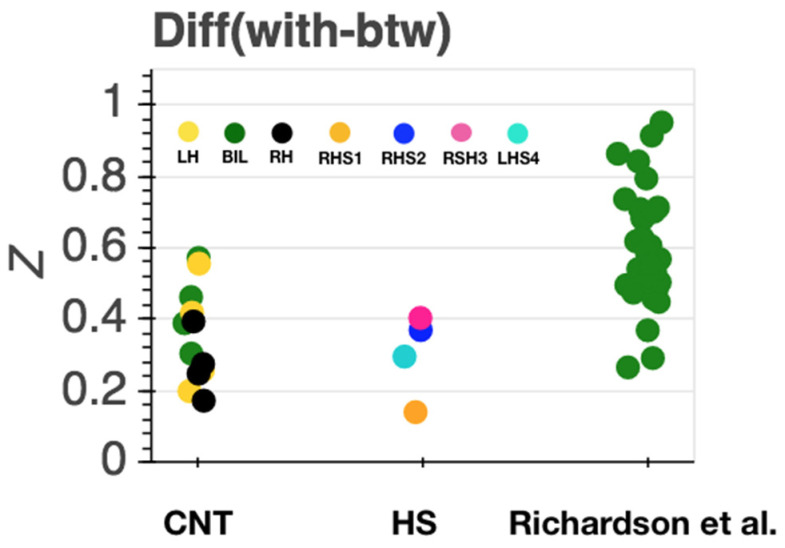
Differences in within and between network connectivity strength per sample CNT (left), HS (middle), comparison sample form Richardson et al. (right) [7]. Each datapoint corresponds to one subject. For CNT, type of ROI is color coded (bilateral: green; LH: yellow; RH: black), for HS, subjects are color coded (RHS1 orange, RHS2: blue, RHS3: pink, LHS4 turquoise), for Richardson et al. comparison data, all data are from bilateral (green) ROIs per subject.

Second, we compared the inter-region correlation among ROIs within and between networks in the remaining intact hemisphere of HS participants. As seen in the comparison participants, all four HS participants exhibited functional separation of the ToM and PAIN networks, with stronger correlation of extracted time series responses in brain regions belonging to the same network (see Figure 3d, 4 middle) and weaker correlation between networks. This pattern was evident for all subjects, with either left or right hemispherectomy. However, correlations were globally elevated (within and between ToM and PAIN networks) for RSH1 (see Figure 4), but the relative within subject difference of the within minus between network regions remained at levels comparable to the other HS participants (see Figure 5).

Third, we assessed how regions of the two social brain networks (PAIN and ToM) in comparison subjects were correlated during movie watching when we restricted inter-region correlation to ROIs exclusively in one hemisphere (see Figure 3b,c). Note that this is only an artificial restriction when analyzing extracted time series since actual network interactions were not anatomically restricted in these healthy brains as they were in the individuals with hemispherectomy. For the left hemisphere (Figure 3c), inter-region correlation analyses’ outcomes remained largely similar to the bilateral ROI set: stronger within than between network correlation across all four comparison subjects. For the right hemisphere (Figure 3b), dorsal and middle MPFC correlations were weaker than correlations for these in the original set of ROIs (Figure 3a), particularly for comparison subjects one, two and three. This pattern (weaker dorsal and middle MPFC correlations) was not evident in the participant who had only a right hemisphere (LHS4, Figure 3d), suggesting that the residual right hemisphere was performing more similarly to intact networks in comparison subjects. In summary, interactions of regions in networks engaged in thinking of others internal and physical states remained, overall, strikingly similar in patients with only one intact cerebral hemisphere.

## 4. Discussion

We have investigated socioemotional functioning and functional brain network organization in a rare population of four individuals with only one intact cerebral hemisphere. At the behavioral level, these individuals showed a strikingly compensated level of overall cognitive functioning. Moreover, they did not evidence clinically impaired social-emotional processing (e.g., consistent with autism spectrum disorders) on a brief battery of standardized self-report and behavioral assessments (cf. Table 3). Cognitive and socioemotional abilities and behavior appeared appropriate in the participants’ presentation and interaction with the experimenter during testing, which was essentially normal except for mild–moderate motor impairments consistent with their lesion (contralateral hemiparesis). At the neural level, activation patterns while watching a movie with socioemotional content showed a relatively preserved dissociation between two social brain networks in the intact hemisphere that are usually distributed across both hemispheres in healthy people. The findings show a remarkable degree of functional neural reorganization in social brain networks, and concomitant compensated behavior, given that the patients have lesions encompassing essentially an entire cerebral hemisphere.

We have leveraged pre-existing work on regions and networks of the brain processing different types of social information, which showed a functional dissociation of brain networks underlying the ability to make social inferences about two types of internal states: other people’s state of mind (e.g., emotions, beliefs, intentions) and the state of their body (e.g., pain, hunger, thirst). While not always entirely mutually exclusive (e.g., grieving a loved one involves the feeling of visceral pain), multiple neuroimaging studies suggest a respective division of labor at the brain network level. The coherence of each network, and the division of labor between them, was quantified by the strength of the within network and between network correlation in the time course of brain activation evoked by our movie stimulus, as in prior studies ([7,32,63]). These neural signatures have been shown to arise early in development and are functionally distinct at age 3 with continuing functional specialization into late childhood [7].

Our study included two comparison samples: one closely matched on demographic characteristics, cognitive functioning, similar scanning parameters, and data processing (our four comparison subjects), and a second to provide a wider reference frame from previously published work with the same task and type of analysis (N = 33 from [7], far replicability study [81]). The four comparison subjects’ data suggest a valid functional dissociation of the ToM and pain network, as expected. The level of anti-correlation between the two networks was less pronounced than in the previously published larger sample. These differences could likely be attributed to elevated noise levels in unsmoothed single subject fMRI data. Nevertheless, social brain networks showed the typical overall pattern of stronger within as compared to between network correlations of brain activity. Since the patient sample of interest in this work only had one intact hemisphere, we provided three different types of inter-region analysis as comparisons: (i) comprising all regions from the two networks (including lateral and midline regions, see Figure 2); (ii) comprising all regions from the left hemisphere with complete lateral ROIs and midline ROIs containing only voxels from the left hemisphere; (iii) comprising all regions from the right hemisphere with complete lateral ROIs in combination with midline ROIs containing voxels mirrored from the left hemisphere to account for small numbers of voxels in right medial prefrontal cortex ROIs. Within and between network correlations were at expected levels for extracted time series for i and ii. For iii, the right hemisphere dorsal and middle MPFC ROI correlations were weaker, particularly for comparison subjects CNT1, CNT2 and CNT3. Given that the original reference location of the MPFC ROIs was more left lateralized, that finding is not surprising. The comparison subjects have two intact hemispheres and therefore lower within network correlation in altered ROIs would be expected, at least to some degree. Along the same lines, the subject-wise difference between networks (Figure 5) was lowest for right hemispheric ROIs in the comparison sample. Interestingly, this pattern (weaker dorsal and middle MPFC correlations) was less evident in LHS4 (Figure 3d) the participant with left hemispherectomy. One likely interpretation is that the residual intact right hemisphere reorganized towards network components that matched those in comparison subjects. Nevertheless, there was no prominent difference in strength of correlation within as compared to between network for left and right hemispherectomy participants. All participants showed the typical distinct network responses to socioemotional stimuli with stronger within than between network synchronization. Notably, RHS1 presented with generally the strongest within *and* between network correlations. Regardless of the overall elevated synchronization across social brain networks, stronger within than between regional correlation was evident (Figure 4 and Figure 5, orange). It is unlikely that increased levels of head motion (sometimes leading to spurious correlation) caused higher synchronization. RHS1 showed the least average amount of head motion while watching the movie in the scanner among all participants. More likely, the relatively stronger within and between network correlation might represent compensatory strengthening of unilateral connections, as previously reported in rsfMRI ([11]). This interpretation would be consistent with the fact that RHS1 also had hemispherectomy at the earliest age in our sample (Table 1), permitting maximal reorganization.

The age at which the hemispherectomy was performed varied largely across the individuals (see Table 1). An open question remains how the stage of development at epilepsy onset and resection, respectively, affects functional reorganization of brain networks and behavioral compensation. Seizure onset occurred not before late childhood in RHS2, RHS3 and LHS4. RHS1 had the hemispherectomy surgery at 3 months of age, with seizures present shortly after birth. In stark contrast, the other two right hemispherectomy cases had relatively late final resections at 20 and 15 years of age. It should be noted that hemispherectomy is typically performed only after years of otherwise unsuccessful pharmacological treatments. Thus, the onset of seizures, generally, substantially precedes the resection, and reorganization may already begin in this time window. If we take only the age of onset of surgery, however, all hemispherectomy cases—except RHS1—should have achieved some degree of functional network dissociation, given prior findings [7,63]. Nevertheless, the typical networks include both midline as well as bilateral regions, and thus reorganization of those network components that were in the resected hemisphere would still be necessary post resection.

Watching naturalistic movies has become a powerful tool when studying functional brain organization, especially for developmental and atypical populations [39,82]. It is possible that additional dysfunctional features of network configurations across the whole brain (or whole hemisphere) that we missed in the present study could be revealed by using a task in the scanner directly involving a behavioral response from subjects. On the one hand, task-fMRI data can have the benefit of closely linking the brain with behavior [83]. On the other hand, passive viewing has the advantage of avoiding confounding activation differences that arise merely from different task performances. If there is no active task, differences in brain activation and network organization are more likely related to differences in stimulus processing and cognition, as opposed to performance differences.

There are more specific models of hemispheric specialization for processing emotional information that we did not directly test in the present study. Within the social domain, prominent asymmetric models of hemispheric specialization focus on emotional valence. Specifically, the *valence hypothesis* posits that valence processing is associated with lateralized brain networks (left lateralized for positive and right lateralized for negative valence) [84,85,86], whereas the *right hemisphere hypothesis* argues for right lateralized dominance for all emotion processing [45,87,88,89,90]. Both of these models have received support from studies of patients with circumscribed lateralized brain lesions. While the current work provides conceptual support for intact general social processing in hemispherectomy patients, the present data do not directly speak to these hypotheses. The pain network has been reported more closely related to negative valenced states (most prominently represented in its name, the pain network), while the ToM network is involved in processing mental state information that could be either positive or negative. Comparing these two networks, thus, does not permit a direct comparison of positive vs. negative valence processing. Nevertheless, our data argue that the right hemisphere cannot be essential for socioemotional processing in any rigidly innate way, given the patient brain reorganization, observed social behavior, and socioemotional test scores suggesting at least some level of compensation.

This study has several limitations. First, this study is a multiple case study of rare individuals, with the expected limitations of a small N. In addition, our sample was highly selected for research purposes limiting our sample size further: all participants were required to have complete anatomical or near-complete functional hemispherectomy, normal intellectual functioning and ability to provide informed consent, ability to complete a comprehensive battery of sociocognitive assessments, and ability to participate in an MRI scan and lie still in the scanner for a significant time. While case studies offer a unique opportunity to assess causal contributions of brain regions (in this study even whole hemispheres) to cognitive processes, the small sample size limits generalization of findings to larger samples and adequate statistical comparison to a comparison sample. Thus, our study applied the logic of case studies, presenting our data as a multiple case study, without group comparisons. Notably, RHS1 stands out from all other subjects in age at surgery and in the overall strengths of the correlations (Figure 4). Although once again the correlation structure reflects the same network organization seen in healthy comparison subjects, it is important to emphasize the heterogeneity of these cases—a topic that will require considerably larger samples in future studies to fully characterize. Second, we have not registered individual subjects’ data to a common template space, but instead have registered the template space ROIs into native subject space. We consider this aspect of our study both a strength (since we limit geometric distortion when transforming to different spaces, retaining anatomical specificity) and a weakness (since we cannot compare activations across participants in the same template space). Third, this study included only one, relatively short, passive viewing task. Increasing task demands with an explicit behavioral response might reveal further insights into common (and idiosyncratic) brain responses and allow for a more detailed relation to socioemotional behavior. Additionally, only the left HS participant scored within the average range compared to published norms on all psychological measures in this study, whereas each right HS participant had at least one atypical score (i.e., more than 1.5 standard deviations from the normative mean), suggesting some degree of atypical socioemotional processing after hemispherectomy. While the laboratory assessments used in this study do not capture the challenges of individuals with hemispherectomy in real life, the scores and their presentation were surprisingly compensated compared to what one might expect following such a large resection of the brain. For instance, surgeries removing far less tissue than a hemispherectomy (e.g., [91]), can produce impairments both on laboratory assessment scores and presentation that are striking and further from standardized mean scores than reported here.

Fourth, lateralization of brain networks may further interact with handedness, i.e., typically right lateralized social brain networks may be less lateralized for left-handed individuals (e.g., [92]). One HS participant (LHS4) was left-handed and also showed relatively typical social brain network dissociation. Given the small sample size, we cannot assess the effect of handedness and lateralization further within the context of this study. Importantly, we do not combine the left-handed subject with the others in any group-based analysis (as is also typically not done in neuroimaging studies of healthy individuals).

## 5. Conclusions

Four individuals with hemispherectomy showed strikingly intact general cognitive as well as varying levels of socioemotional processing on the behavioral level. We have assessed two social brain networks underlying socioemotional processing. Comparison of the functional neuroimaging data with two separate groups of healthy comparison subjects suggests that reorganization at the network level in social brain regions of the remaining intact hemisphere was sufficient to delineate these networks. Taken together, the findings argue for a remarkable degree of cortical reorganization that is possible in networks subserving socioemotional processing.

## Figures and Tables

**Table 1:** Demographic participant information. Hem, hemisphere removed; Etiol, etiology; HS, hemispherectomy; Age HS, age of final hemispherectomy surgery; Onset, onset of epileptic seizures; Hand, handedness; FSIQ, Full Scale Intelligence Quotient; VCI, Verbal Comprehension Index; POI, Perceptual Organization Index; R, right; L, left; CD, Cortical Dysplasia; RS Rasmussen’s encephalitis; PNS, perinatal stroke; m, months; y, years; M, male; F, female; C, comparison; na = not applicable.

Case	Hem	Etiol	Onset	Age HS	Sex	Hand	Age	FSIQ	VCI	POI
RHS1	R	CD	birth	3m	M	R	22	80	91	72
RHS2	R	RS	11y	20y	M	R	23	105	109	93
RHS3	R	PNS	8y	15y	F	R	21	96	101	86
LHS4	L	PNS	3y	6y	F	L	26	95	101	95
CNT1	na	na	na	na	M	R	26	94	99	92
CNT2	na	na	na	na	F	R	24	100	104	96
CNT3	na	na	na	na	F	L	28	100	106	94
CNT4	na	na	na	na	M	R	24	101	105	96

**Table 2:** Head motion per subject. Temporal mean framewise displacement (FD) in millimeter per case and run, as well as mean over runs, where applicable.

Case	Temporal Mean FD (mm)		
	run1	run2	mean(run1,run2)
RHS1	0.10	n/a	n/a
RHS2	0.13	0.20	0.16
RHS3	0.15	0.11	0.13
LHS1	0.23	0.12	0.17
CNT1	0.30	n/a	n/a
CNT2	0.14	n/a	n/a
CNT3	0.11	n/a	n/a
CNT4	0.16	n/a	n/a

**Table 3:** Z-scores on Self-Report Questionnaires and an Emotion Processing Task: For all scores, higher scores indicate more of the trait being assessed. * greater than 1.5 standard deviations from the mean. MSCEIT, Mayer-Salovey-Caruso Emotional Intelligence Test; Exp, Experiencing; Rea, Reasoning; PANAS, Positive and Negative Affect Scales; Pos, Positive; Neg, Negative; STAI, State-Trait Anxiety Inventory; SRS-2, Social Responsiveness Scale-2 Adult Self-Report; EQ, Empathizing Quotient; SQ, Systemizing Quotient.

Case	MSCEIT Exp	MSCEIT Rea	PANAS Pos	PANAS Neg	STAI State	STAI Trait	SRS-2	EQ	SQ
RHS1	0.61	−0.38	2.21 *	−0.75	−1.00	−0.50	−0.50	1.64 *	1.40
RHS2	−0.45	0.57	−0.37	−1.05	−1.20	−1.20	−1.00	2.24 *	0.30
RHS3	0.28	−0.16	−1.59 *	−1.05	−0.10	1.00	0.80	−1.15	−0.40
LHS4	−0.42	−0.66	−1.32	−0.01	1.40	1.10	1.40	−0.27	1.16
CNT1	na	na	na	na	na	0	0.30	−1.38	−0.79
CNT2	0.09	−0.47	na	na	na	1.00	1.40	0.27	0.54
CNT3	0.03	0.36	na	na	na	1.30	0.30	0.71	0.02
CNT4	na	na	na	na	na	2.00*	1.30	−0.52	−0.53

## Data Availability

The data presented in this study are in part openly available in [https://nda.nih.gov/edit_collection.html?id=2417, accessed on 15 July 2021, https://openneuro.org/datasets/ds000228, accessed on 15 July 2021,); data from hemispherectomy subjects are not publicly available due to privacy restrictions.
